# Isoniazid-induced acute psychotic episode in a child with lymph nodes tuberculosis: case report and literature review

**DOI:** 10.1192/j.eurpsy.2023.1082

**Published:** 2023-07-19

**Authors:** H. Belhadga, Z. Elmaataoui, H. Kisra

**Affiliations:** Hôpital psychiatrique universitaire arrazi de sale, Hôpital psychiatrique universitaire arrazi de SALE, Sale, Morocco

## Abstract

**Introduction:**

Tuberculosis (TB) is a bacterial infection caused by Mycobacterium tuberculosis and is a major public health problem in Morocco. The World Health Organization (WHO) recommends a four-drug combination therapy for two months (isoniazid (INH), rifampicin, pyrazinamide and ethambutol), followed by two drugs (INH and rifampicin) for 4 months, as first-line treatment for newly diagnosed pulmonary TB in children and adults. This regimen is generally considered effective, safe, and cost-effective. However, adverse effects and drug interactions often complicate the treatment of TB. Isoniazid is associated with 32% of adverse events, of which 1.9% are psychiatric.

**Objectives:**

focus on drug-induced etiologies of acute psychotic symptoms make the diagnosis of isoniazid-induced psychosis report the literature on the management of this condition. focus on drug-induced etiologies of acute psychotic symptomsmake the diagnosis of isoniazid-induced psychosis report the literature on the management of this condition.

**Methods:**

The patient and guardians were interviewed to obtain information after their consent. Data from the patient profile forms and laboratory test reports were evaluated. Causality assessment was done by the WHO scale and the Naranjo scale. Oral informed consent was obtained from the patient and parents.

**Results:**

We report a case of acute psychosis in a child with a temporal sequence strongly in favor of INH-induced psychosis. Treatment with risperidone at an appropriate dose improved the symptomatology while waiting for the end of the antituberculosis protocol. As soon as the isoniazid was stopped, there was a clear return to the child’s premorbid state.

**Conclusions:**

The acute onset of psychotic symptoms in a patient taking isoniazid should lead to suspicion of this psychiatric side effect and prompt intervention, involving discontinuation of isoniazid and/or a trial of an antipsychotic.

As protective measures, the authors suggest adjusting the dose of isoniazid to weight, possibly performing a genetic test if slow acetylation is suspected, and closely monitoring patients with a favourable background.

The acute onset of psychotic symptoms in a patient taking isoniazid should lead to suspicion of this psychiatric side effect and prompt intervention, involving discontinuation of isoniazid and/or a trial of an antipsychotic.As protective measures, the authors suggest adjusting the dose of isoniazid to weight, possibly performing a genetic test if slow acetylation is suspected, and closely monitoring patients with a favourable background.

**Disclosure of Interest:**

None Declared

